# Perceptions of Others' Political Affiliation Are Moderated by Individual Perceivers' Own Political Attitudes

**DOI:** 10.1371/journal.pone.0095431

**Published:** 2014-04-29

**Authors:** John Paul Wilson, Nicholas O. Rule

**Affiliations:** Department of Psychology, University of Toronto, Toronto, Ontario, Canada; Tilburg University, Netherlands

## Abstract

Previous research has shown that perceivers can accurately extract information about perceptually ambiguous group memberships from facial information alone. For example, people demonstrate above-chance accuracy in categorizing political ideology from faces. Further, they ascribe particular personality traits to faces according to political party (e.g., Republicans are dominant and mature, Democrats are likeable and trustworthy). Here, we report three studies that replicated and extended these effects. In Study 1a, we provide evidence that, in addition to showing accuracy in categorization, politically-conservative participants expressed a bias toward categorizing targets as outgroup members. In Study 1b, we replicate this relationship with a larger sample and a stimulus set consisting of faces of professional politicians. In Study 2, we find that trait ascriptions based on target political affiliation are moderated by perceiver political ideology. Specifically, although Democrats are stereotyped as more likeable and trustworthy, conservative participants rated faces that were categorized as Republicans in Study 1a as more likeable and trustworthy than faces categorized as Democrats. Thus, this paper joins a growing literature showing that it is critical to consider perceiver identity in examining perceptions of identities and traits from faces.

## Introduction

People can be quite accurate at extracting a number of seemingly concealable social identities from facial information alone. Unlike categorizations based on race, sex, and age, other group memberships (e.g., sexual orientation, religion, and political affiliation) are quite perceptually ambiguous. Nevertheless, perceivers reliably exhibit above-chance accuracy for distinguishing members of these groups [1]. These categorizations, at least for some dimensions, seem to be driven by the perception of subtle cues that may differ between groups [2]. Accordingly, much of the existing research on accuracy in categorizing ambiguous group members has focused on the target, leaving much unknown about the perceiver's contribution to these judgments.

For example, studies have found that participants can accurately distinguish political affiliation based on photos of faces [3], [4], [5], [6] [7], [8]. Furthermore, Rule and Ambady [4] found that these effects seem to have been driven by traits attributed to the faces; specifically, power (a composite of ratings of dominance and facial maturity) and warmth (a composite of ratings of likeability and trustworthiness). Republican faces were perceived as more powerful than Democrats and, to the extent that a face was perceived as powerful, it was more likely to be categorized as Republican. On the other hand, the warmer a face was perceived, the more likely it was to be categorized as a Democrat. Samochowiec et al. [6] reported similar findings: perceived dominance partially accounted for the relationship between targets' perceived and actual political ideology in Europe.

Other studies have also found relationships between facial traits and perceived political ideology. For example, Berggren, Jordahl, and Poutvaara [9] found that conservative politicians in Finland were more attractive than candidates on the political left. This is similar to a result reported by Bull and Hawkes [10] in which politicians judged to be conservative were more attractive, intelligent-looking, and of higher social class than those judged to be more liberal. These judgments can have electoral consequences. Though Bull et al. [11] found that these ratings did not correspond to vote share, Olivola et al. [8] found that politicians in conservative geographic areas tend to benefit in terms of electoral outcomes if they have a conservative-looking appearance.

Although the extant research has provided important information about factors that may underlie categorizations of faces according to political party affiliation, it may be limited in some critical ways. For example, the effects described by Rule and Ambady [4] result from a focus primarily on the target in isolation from the perceiver. It is important to note that this research draws from ideas based in an ecological theory of perception [12] adapted to theories of ecological social perception [13], [14]. These theories argue that faces signal certain things to perceivers about what the target may afford. A target may appear, for instance, more or less trustworthy or dominant [15], [16], which may lead the perceiver to trust or fear that person, accordingly. However, it is likely that the accuracy of categorizing ambiguous targets is driven at least in part by the perceivers' identities, dispositions, or states. For example, it is known that perceptions of ambiguous group members may be influenced by perceivers' attitudes toward the groups [17] or exposure to members of the group [18]. Brambilla et al. [18] found that heterosexual perceivers who had more experience with gay men were better at categorizing gay faces. Similarly, it is plausible that categorizations and trait ascriptions of Democrat and Republican faces may differ based on the political ideology of the perceiver/judge. There does exist some evidence that perceiver identities and ideologies influence categorizations and judgments based on political affiliation. Jahoda [5], for instance, found that people categorized faces as political ingroup or outgroup members largely as a function of likeability. More recently, Chiao et al. [19] found that perceiver gender influences ratings towards male and female politicians.

Target political affiliation may interact with perceiver identity in another important way. In addition to accurate perceptions of political ideology, Samochowiec et al. [6] also found that participants were more likely to classify faces as outgroup members than as ingroup members. These findings are consistent with a more general ingroup overexclusion effect [20], [21]. The ingroup overexclusion effect is thought to be a result of motivated social cognitions related to social identity, such that people tend to be protective of the ingroup. As a result of this protectiveness, perceivers may show a default bias toward categorizing others as outgroup members. This may be the case especially when groups are perceptually ambiguous. For example, Blascovich, Wyer, Swart, and Kibler [22] observed an ingroup overexclusion effect for racially ambiguous targets, and Castano et al. [21] found that Northern Italians (who had strongly identified as such) were more likely to exclude ambiguous targets that had a mix of Northern and Southern Italian features.

The current work represents another attempt to incorporate perceiver identities into understanding the legibility of target political affiliation from faces. The work conducted by Rule and Ambady [4], for example, was limited in that it employed a sample that was demographically liberal in an area where Democratic politicians tend to be highly favored by the public at large. Findings suggesting that Republicans are perceived as more powerful and Democrats as more warm may be the result of a biased sample. In fact, Olivola et al. [8] failed to find a relationship between perceived political party and judgments of traits such as honesty and dependability with an Internet sample from the US. Aggregate perceptions across diverse sets of perceivers may not correspond with target political party, highlighting the importance of the possible role that individual differences in perceiver identities may exert upon judgments. The current research therefore included participants across the political spectrum from an Internet community sample. This made it possible to investigate how categorizations and trait ascriptions might differ based on perceivers' political orientation.

In three studies, we investigated how perceivers' political ideology influenced their perceptions of faces from different political parties. In each study, rather than use a single-item scale or self-categorization of party membership, we asked participants to complete a validated scale measuring political values: the McClosky political conservatism scale [23], [24]. This allowed us to more precisely measure a continuous relationship between political ideology and perceptions of targets according to perceived party. We used the revised version of the scale reported by McClosky and Bann [24], including both the 19-item Classical Conservatism scale and seven items addressing social welfare issues, for a total of 26 items. This 26-item version has been used as a broad measure of conservatism-liberalism [25], [26]. For each item on the scale, participants can select either the conservative response or the liberal response (or neither).

In Study 1, we tested the relationship between perceivers' political leanings and their categorizations of targets as Democrats and Republicans. In addition to expecting to replicate past results showing above-chance accuracy overall, we expected perceiver ideology to influence categorizations. Based on prior research on social groups and categorization [20], we predicted that participants would tend to *overexclude* targets from the ingroup. In other words, we predicted that conservatives would show a default tendency to categorize faces as Democrat outgroup members, and that liberals would show a default tendency to categorize faces as Republican outgroup members. Thus, we expected individuals' political beliefs to bias their categorizations in a way that guards their ranks against potential adulteration by outgroup members, consistent with theories of ingroup overexclusion [20].

Further, and more important, in Study 2 we extended beyond previous work to predict that perceivers' political leanings would moderate how personality traits would be differentially ascribed to Democrat and Republican faces. As past work sampling participants from a highly politically liberal population showed a relationship between Democrats and warmth, we therefore tested whether this is a function of the targets (i.e., warmer faces are universally more likely to be seen as Democrats) or of the perceivers (i.e., warmer faces are more likely to be seen as ingroup members for both conservatives and liberals). The latter result would be consistent with previous reports showing that voters tend to associate likeability and warmth with their favored political candidates, which tend to be those who share their political beliefs [5], [27], [28]. In other words, we hypothesized that conservative participants would not show the previously described tendency to rate Democrat faces as more likeable and trustworthy. Rather, these participants were expected to see *Republican* faces as more likeable and trustworthy—thereby showing evidence of ingroup favoritism [29]. Moreover, we predicted that these relationships would be mediated by the extent to which a face was likely to be categorized as Republican. Thus, conservative perceivers should see faces that belong to actual Republicans as likeable to the extent that they can be accurately categorized by party affiliation.

## Study 1a

The present study aimed to expand on previous work showing that individuals are able to accurately perceive others' political affiliation [3], [4], [5], [6], [7], [8]. This work has largely but not exclusively (see [5], [8], [19]) focused on the targets of perception, rather than the characteristics of the perceivers making the judgments. Some of these studies asked participants to self-report their affiliation with particular political parties but reported little or no relationship between the participants' political party and their categorizations of the faces [3], [4], [5]. Rather than inquire about party membership, one study asked participants to self-report their political leaning along a continuous scale ranging from conservative to liberal [6].

The present study aimed to expand on this past work by assessing participants' political attitudes with the McClosky political conservatism scale [23], [24]. Like Samochowiec et al. [6], we expected that participants would show an ingroup overexclusion effect whereby participants with higher scores on the conservatism scale would be more likely to categorize targets as Democrats versus Republicans (and, as such, participants on the liberal end of the scale would show the opposite tendency). Although Democrats and Republicans are not isomorphic with liberals and conservatives, the two correspond tightly; see Abramowitz and Saunders (2008) for discussion and Study 1a results for data illustrating this. In addition, the current design uses discrete categorizations of targets into specific political groups (Democrats and Republicans), as opposed to the scalar measure of political behavior used in Samochowiec et al.'s [6] study. Although the method used by Samochowiec et al. [6] allows for greater sensitivity in assessing accuracy, a potential advantage of the dichotomous categorization task here is that it may allow for a more precise estimate of participants' response bias measure than was possible in the preceding work, which required bifurcation of the scale to somewhat artificially divide the targets into two groups.

### Method

#### Ethics statement

This study was approved by the Research Ethics Board at the University of Toronto. All participants provided written informed consent prior to participation in the study.

#### Participants

Forty-six American participants engaged in the experiment via Amazon's Mechanical Turk [30]; two did not complete the experiment and their data were removed from analysis for a total of 44 participants [23 male (52%), *M*_Age_  = 32 years, *SD*  = 11; 35 White (80%), 3 Hispanic (7%), 2 Black (5%), 1 Asian (2%), 3 Multiracial/Other (7%)]. No other participants were removed from analysis for any other reason. The self-reported party affiliation of our participants was as follows: 8 Republican (18%); 11 Democrat (25%); 7 Libertarian (16%); 1 Green (2%); 14 Independent (32%); 3 other (7%).

#### Stimuli

Stimuli were borrowed from a previous study examining judgments of Americans' political party affiliation [4]. Photos consisted of 60 male and female Democrat (*n* = 30) and Republican (*n* = 30) university students from a small liberal arts college in the northeast US. Among Democrats, 15 were male and 15 were female. Among Republicans, 21 were male and 9 were female. All were Caucasian. All of the photos were digitally scanned from the portrait section of the students' senior yearbooks. The students had indicated either the Democrat or Republican student organization among their extra-curricular activities in the yearbook; however, the photos were not taken from club photos or other contexts in which the students' political affiliations would have been salient at the time of photography. Each of the images was cropped to the extremes of the head: top of hair, bottom of chin, and sides of ears. All of the images were grayscale and standardized to be of equal face height while maintaining the aspect ratio of the face. The stimuli are available upon request.

#### Procedure

Participants were instructed that they would be seeing a series of faces presented on their computer's screen and that they were to categorize them as Democrats or Republicans as quickly and accurately as possible, relying on their “gut” instinct. All of the face stimuli described above were presented in random order and participants provided their responses via mouse-click. After categorizing all of the faces, participants were asked to complete the McClosky political conservatism scale and some demographic questions, including the opportunity to self-categorize among several conservative (Republican, Libertarian, Tea), liberal (Democrat, Green), or neither (independent, other) parties. Consistent with reports of the polarization of political parties in the contemporary US, despite diverse self-categorizations outside of the traditional bipartisan split [31], participants' conservatism scores were highly correlated with their categorical party affiliations for the subsets identifying as Democrats or Republicans and more broadly as any of the liberal or conservative parties: all *r*'s>.71.

### Results

Data from Study 1a are available here http://dx.doi.org/10.6084/m9.figshare.967938. Participants' categorizations of the faces as Democrat and Republican were analyzed using the signal detection theory statistics *A*′ and *B*″ to measure sensitivity and response bias, respectively (see Macmillan & Creelman [32] for formulas). *A*′ is a nonparametric measure of recognition accuracy that does not require homogeneous variance and can be calculated when participants exhibit hit or false alarm rates of 1 or 0. *B*″ is a complementary measure of response bias. Correct categorizations of Democrats were counted as hits and incorrect categorizations of Republicans were counted as false-alarms. Because we coded correct categorizations of Democrats as hits, negative *B*″ values represent a tendency to categorize targets as Democrats, whereas positive values represent a tendency to categorize targets as Republicans.

Replicating previous work with an undergraduate laboratory sample [4], participants were significantly more accurate than chance in categorizing the targets' political affiliations: *M*_A_′  = .63, *SD*  = .12, *t*(43)  = 7.15, *p*<.001, *Cohen*'*s d* = 1.07. Overall, participants showed no significant bias toward categorizing targets as Democrats or Republicans (*M*_B_″  = −.03, *SD*  = .15), *t*(43)  = 1.49, *p* = .14, *Cohen*'*s d* = 0.22.

Critically, participants' response bias was significantly correlated with their levels of political conservatism. Following the analyses of Peterson-Badali et al. [25], we calculated the percentage of conservative responses chosen by participants on the McClosky scale: *M* = 39%, *SD*  = 22%; inter-item reliability Cronbach's α = .86. It should be noted that this scale also provides an index of liberalism but, as these were strongly negatively correlated with the percentage of conservative responses (*r* = −.77), all results in the present work are discussed in terms of conservatism. Looking at the relationship between ideology and response bias (*B*″), we observed a greater tendency for participants to categorize targets as Democrats as a function of their endorsement of conservative items on the scale, suggesting an ingroup-overexclusion effect [14]: *r*(42)  = −.29, *p* = .052—though this correlation did not reach conventional levels of statistical significance (i.e., *p*<.05). Conservatism was not correlated with participants' accuracy (*A*′): [*r*(42)  = −.14, *p* = .36], conceptually replicating past work in which the political affiliation of perceivers did not correlate with accuracy in categorizing US politicians [33] (those authors also examined response bias, but did not report as to whether it was associated with political affiliation).

## Study 1b

Study 1b was designed to replicate Study 1a while addressing a few possible shortcomings. First, the relationship between conservatism and response bias did not reach conventional levels of significance, perhaps due to issues with statistical power. Here, we therefore increased the sample size in order to investigate whether the ingroup overexclusion effect is reliable. Next, Study 1a used a limited stimulus set of non-politicians. Here, we used a larger set of faces, this time of professional American politicians, in order to establish the generalizability of the observed relationships. Finally, faces were not blocked according to target sex in Study 1a, above. As female politicians may be more likely to be Democrats [34], presenting the faces in uniform blocks by target sex might reduce participants' ability to rely on sex to infer political party. Thus, Study 1b used a design consisting of blocks by target sex.

### Method

#### Ethics statement

This study was approved by the Research Ethics Board at the University of Toronto. All participants provided written informed consent prior to participation in the study.

#### Participants

One hundred twenty-one American participants engaged in the experiment via Amazon's Mechanical Turk [30] [56 male (46%), *M*_Age_  = 35 years, *SD*  = 12; 87 White (72%), 7 Hispanic (6%), 8 Black (7%), 10 Asian (8%), 9 Multiracial/Other (7%)]. No participants were removed from analysis for any reason. The self-reported party affiliation of our participants was as follows: 21 Republican (17%); 57 Democrat (47%); 6 Libertarian (5%); 4 Green (3%); 16 Independent (13%); 17 other (14%).

#### Stimuli

Stimuli were once again borrowed from a previous study examining judgments of Americans' political party affiliation [4]. Here, however, we used photos of professional politicians who ran for the US Senate in 2004 and 2006. Each photo was cropped to the extremes of the targets' heads (top of head, bottom of chin, sides of hair or ears), converted to grayscale, and standardized for size. Ethnic minority targets were not used in this study. Daniel Akaka, a non-White Democrat, was mistakenly included in the stimulus set, but analyses excluded him. In total, we used 115 target faces: 58 Democrats (*n* = 15 women) and 57 Republicans (*n* = 5 women). All stimuli are available as supplemental files here http://dx.doi.org/10.6084/m9.figshare.967942.

#### Procedure

The main procedure was identical to Study 1a with the exception that the faces were presented randomly within randomly-ordered blocks that were uniform in gender. After completing the face categorization task and the McClosky scale, participants were asked to indicate their political party identification in an open-ended format. We also asked participants to list any faces that they had recognized. We performed our analyses on the complete dataset, as well as after removing individual trials in which a participant recognized a face (0.68% of all trials, in total), and the results did not change appreciably; we therefore report the full data below.

### Results

Data from Study 1b are available here http://dx.doi.org/10.6084/m9.figshare.967939. As in Study 1a, participants' categorizations of the faces as Democrat and Republican were analyzed using *A*′ and *B*″ to measure sensitivity and response bias, respectively. Correct categorizations of Democrats were counted as hits and incorrect categorizations of Republicans were counted as false-alarms. Replicating Study 1a, participants were significantly more accurate than chance in categorizing the targets' political affiliations: *M*_A_′ * = *.54, *SD*  = .09, *t*(120)  = 5.12, *p*<.001, *Cohen*'*s d* = 0.47. Unlike Study 1a, participants showed a slight bias towards categorizing targets as Republicans (*M*_B_″  = .009, *SD*  = .04), *t*(120)  = 2.39, *p* = .02, *Cohen*'*s d* = 0.19.

As in Study 1a, we calculated the percentage of conservative responses chosen by participants on the McClosky scale: *M* = 33%, *SD*  = 21%; inter-item reliability Cronbach's α = .88. Participants in Study 1b were slightly less conservative than in Study 1a. Looking at the relationship between ideology and response bias (*B*″), we saw that participants once again showed a greater tendency to categorize targets as Democrats as a function of their endorsement of conservative items on the scale: *r*(119)  = −.24, *p* = .007. As in Study 1a, conservatism was not correlated with participants' accuracy (*A*′): *r*(119)  = −.12, *p* = .18.

### Discussion

As predicted, participants in both Studies 1a and 1b showed above-chance accuracy in categorizing target faces according to political party affiliation. This replicates past work on the accuracy of judging political orientation from static facial information alone and, notably, does so for target samples of both professional politicians and non-politicians. Thus, party membership is expressed through appearance generally for both politicians and non-politicians, as shown in previous work [4]. Further, we also found that participants showed a tendency to overcategorize targets as outgroup members in both studies. This reinforces previous work showing a similar relationship between estimated response bias and participant political affiliation using targets in European parliaments [6]. Thus, as social identity theory researchers have posited, and as our results confirm, people tend to set a relatively high threshold for categorizing an ambiguous target as an ingroup member. This is sensible, as one way to maintain a positive social identity is to be cautious about whom one allows into the ingroup [20], [21].

This result additionally suggests that the encoding of ambiguous social category information may be influenced by social contextual factors [21], [35]. That is, faces transmit a wealth of information, much of which is accurately identified by perceivers. However, this process is influenced not just by target-based affordances, but also by the motivations of the perceiver. Study 2 explored how perceiver identities may also influence the relationship between perceived political party and perceived personality traits.

## Study 2

The results of Studies 1a and 1b showed that perceivers' method of categorizing targets as Democrats and Republicans was influenced by their personal political beliefs. As individuals endorsed more conservative values, they were significantly less likely to think that targets were also conservatives, suggesting an ingroup overexclusion effect [20]. Previous research on perceptions of Democrats and Republicans reported that members of the two groups were associated with different personality traits [4]. Specifically, Republicans were perceived to be significantly more dominant and facially mature than were Democrats and this difference partially accounted for accuracy in judging the targets' political affiliation. Interestingly, that work also found that—irrespective of actual political group membership—targets consensually believed to be Democrats were perceived as high in likeability and trustworthiness, whereas targets consensually believed to be Republicans were perceived as high in dominance and facial maturity. Given that the participants in these studies were undergraduates at a relatively politically liberal university in the Boston metropolitan area, we wondered whether the relationship between perceived political party and perceived personality traits might be moderated by individual variation in political beliefs. Thus, in Study 2, we asked an online community sample of participants to assess the personality traits of the same targets and related their judgments to a measure of their political attitudes. We predicted that participants would rate Republican faces as more likeable and trustworthy to the extent that they report more conservative attitudes.

### Method

#### Ethics statement

This study was approved by the Research Ethics Board at the University of Toronto. All participants provided written informed consent prior to participation in the study.

#### Participants

Fifty-nine American participants engaged in the experiment via Amazon's Mechanical Turk; twelve did not complete the experiment and their data were removed from analysis for a total of 47 participants [21 male (45%), *M*_Age_  = 35 years, *SD*  = 13; 37 White (79%), 1 Hispanic (2%), 5 Black (11%), 4 Asian (9%); 19 Democrat (40%), 5 Republican (11%), 3 Green Party (6%), 2 Libertarian (4%), 13 Independent (28%), 5 Other(11%)].

#### Stimuli

Stimuli were the same as in Study 1a.

#### Procedure

Participants were instructed that they would be seeing a series of faces presented on their computer's screen and that they were to rate them on various traits as quickly and accurately as possible, relying on their “gut” instinct. Participants viewed blocks of faces organized by trait such that they saw each face in random order within each block and rated it on one of either dominance (rated from 1 =  “Submissive” to 7 =  “Dominant”), facial maturity (rated from 1 =  “Babyish” to 7 =  “Mature”), likeability (rated from 1 =  “Not at all likeable” to 7 =  “Very likeable”), or trustworthiness (rated from 1 =  “Not at all trustworthy” to 7 =  “Very trustworthy”) before moving on to the next trait block; participants provided their responses via mouse-click and all of the target faces were presented in each block. Trait-blocks were presented in random order. Once participants had completed each of the four blocks, they were asked to complete the 26-item McClosky political conservatism scale and some demographic questions. No mention of politics or political parties was made at any point during study recruitment or execution; thus, participants were ostensibly rating faces on personality traits and nothing more.

### Results

Data from Study 2 are available here http://dx.doi.org/10.6084/m9.figshare.967940.

#### Replication of past work

As in the previous studies, the sample was more inclined to endorse liberal versus conservative values: on average, participants endorsed only 32% (*SD*  = 18%) of the conservative statements on the McClosky scale (inter-item reliability Cronbach's α = .77). Similar to previous work [4], which analyzed the data with targets rather than participants as the unit of analysis (here, all inter-rater reliabilities Cronbach's α's>.92), Republican targets were perceived as significantly more dominant [*r*(58)  = .34, *p* = .007] and facially mature [*r*(58)  = .28, *p* = .03] than were Democrat targets but showed no differences for likeability [*r*(58)  = −.14, *p* = .29] or trustworthiness [*r*(58)  = −.17, *p* = .19]. The lack of a significant relationship between targets' party affiliation and ratings of their likeability and trustworthiness is inconsistent with some past research [4], though the correlation coefficients are in the expected direction. This lack of a statistically significant correlation may reflect the fact that this sample was likely more conservative than some samples used in past work. In addition, the extent to which targets were perceived as looking Republican (based on the categorizations made in Study 1a) was positively associated with how dominant [*r*(58)  = .40, *p* = .002] and facially mature [*r*(58)  = .31, *p* = .02] the targets were rated, and was negatively associated with how trustworthy [*r*(58)  = −.25, *p* = .05] and likeable [*r*(58)  = −.23, *p* = .08] they were rated, though the latter correlations were only marginally significant. Thus, the present data replicated the relationships reported in previous research [4].

#### Main analysis: Perceiver political beliefs and trait ratings

Of more pertinence to the central question, however, accounting for individual differences in participants' political beliefs substantially moderated these relationships. As we were primarily interested in the relationship between participants' political beliefs and their perceptions of the targets according to political party, we conducted the analyses with the participants as the unit of analysis. Hence, we correlated each participant's ratings on each trait with a dummy-coded vector in which Democrat targets were coded as 0 and Republican targets were coded as 1 and then calculated point-biserial sensitivity correlations indexing the extent to which Republican targets were rated higher on each trait by each participant. In other words, we correlated a target's party identification with trait ratings for each participant. After transforming the resultant correlation coefficients into Fisher's *z* scores, we then correlated them with the participants' endorsement of conservative beliefs measured by the McClosky scale; the resulting value therefore represented the correlation between each participant's individual level of conservatism and the extent to which the participant rated Democrat and Republican targets differently for each trait. Some participants provided the same rating for all of the faces in a particular block/trait, rendering it impossible to calculate a sensitivity correlation; hence, the degrees of freedom for the correlations between the McClosky conservatism scores and face ratings varied slightly between traits.

The more conservative the participants rated themselves to be, the more likely they were to see Republican targets as both more likeable [*r*(44)  = .32, *p* = .03] and more trustworthy [*r*(43)  = .34, *p* = .02] than Democrat targets. No significant correlations were observed for dominance [*r*(44)  = −.07, *p* = .62] and facial maturity [*r*(45)  = −.15, *p* = . 31]. This suggests that individuals view others who share their political views as more likeable and more trustworthy, which may reflect ingroup favoritism. The null correlation for maturity should perhaps be treated with some caution, as the sample is somewhat small. However, we had no prior reason to predict a relationship between perceived maturity and ingroup affiliation.

To better understand the differences in ratings made to Democrat and Republican targets along these traits and to test the ingroup favoritism hypothesis, we measured the extent to which the perceived political affiliation of the targets might statistically mediate the relationship between actual political affiliation and participants' ratings of the targets' traits as a function of the participants' conservatism. To achieve this, we calculated partial sensitivity correlations for each participant that controlled for the consensus perception of each target's political affiliation, based on the ratings given in Study 1a. We therefore computed the proportion of participants in Study 1a who categorized each target as a Republican, producing a decimal value ranging between 0 and 1 that indexed how “Republican” each target face looked (also used in the replication analyses reported above). We then calculated point-biserial correlations between the targets' actual party membership and the participants' ratings of their likeability and trustworthiness, respectively, while controlling for the degree to which each target looked Republican. We then transformed these partial point-biserial correlation coefficients into Fisher's *z* scores and correlated them with the participants' scores on the McClosky conservatism scale.

After controlling for the extent to which each target looked Republican, the relationship between participants' conservatism and the degree to which they viewed Republicans as more trustworthy than Democrats was reduced in size and no longer significant: *r*(43)  = .20, *p* = .19. Similarly, the relationship between participants' conservatism and the degree to which they viewed Republicans as more likeable than Democrats was also reduced in size and yielded a correlation that was only marginally significant after controlling for the extent to which each target looked Republican: *r*(44)  = .29, *p* = .054. These findings suggest that more conservative participants' distinction between conservative versus liberal others as more likeable and more trustworthy may rely, in part, on the legibility of the targets' political group membership. Thus, targets who look more Republican and less Democrat may be liked and trusted more by more conservative participants (see also [8]).

### Discussion

As we predicted, Study 2 showed that perceiver political ideology moderates the relationship between perceptions of target party affiliation and trait ascriptions. These results are somewhat consistent with past research in which perceptions of political party membership occurred largely as a function of participants' own political leanings and the favorability of targets based on appearance [5], [8]. We were able to extend upon this past work by statistically demonstrating that favorable personality ratings (such as trustworthiness and likeability) may have been ascribed to the extent that a target was more likely to be categorized by his or her political affiliation. In other words, the legibility of targets' political affiliation may drive favorable ratings for ingroup faces, though it may have done so without perceivers' explicit awareness.

Importantly, these results were obtained by examining the relationship between political categorizations made by one sample of participants and ratings made by a separate sample. As such, participants are not merely ascribing favorable traits to targets that they have personally identified as ingroup members or expressing some autocorrelative effect. Past work in this domain, on the other hand, has more directly linked particular perceivers' political attitudes with the same perceivers' judgments of faces [5] (but see [8]). Furthermore, we found it intriguing that target political affiliation plays a role in trait judgments even when perceivers have not been oriented to the political nature of the task (i.e., they do not realize that these faces have been identified as affiliating with one or another political party). These results are similar to research by Olivola et al. [8] in which candidates who look Republican are more likely to receive the votes of Republican voters. Here, however, we have used a continuous measure of political ideology, so our results are not exclusive to conservative participants – rather, the more conservative one is, the more likeable and trustworthy Republican faces look; and the less conservative one is, the more likeable and trustworthy Democrat faces look. More centrally, this study serves to qualify existing research on perceived differences between Republican and Democrat faces by showing that these differences are moderated by perceivers' ideology. What is perceived as likeable and trustworthy, then, is a function of the interplay between target and perceiver.

## General Discussion

Perceptions of political group membership appear to be influenced by perceivers' own political leanings. First, we replicated past research demonstrating that people can accurately categorize others by political affiliation [3], [4], [5], [6] [7], [8]. Second, we observed a propensity for categorizing faces as outgroup members, consistent with the ingroup overexclusion effect [6], [20]. Finally, we found that perceivers' ascriptions of traits to faces differing in political affiliation are driven by the extent to which they can glean political group membership from faces. This provides an important qualification to previous research finding that Republicans are seen as more dominant and less likeable than Democrats [4]. Here, we found that Republicans may actually be liked and trusted *more* by perceivers, but only to the extent that the perceiver is conservative and the face can be correctly categorized as a Republican. Our results imply that previous work suggesting a more general relationship between party affiliation and facial characteristics was not fully justified [4]. Rather, the current results suggest a more nuanced relationship between target characteristics, perceiver characteristics, and facial judgments.

This work joins other recent papers in finding that that the basic tendency toward accuracy in categorization and stability in trait inferences for members of ambiguous groups is malleable to the social context [18], [36], [37], [38], [39], [40]. In light of this previous research, this is quite sensible. We found that perceivers ascribed favorable trait ratings to targets who were apparently ingroup members. This is perhaps not surprising, as people tend to show ingroup favoritism across a host of domains, a phenomenon that extends to face perception [41]. For example, other research has shown that racially ambiguous faces are more likely to be categorized as outgroup members than ingroup members when the faces display anger [42]. Further, using data-driven reverse correlation methods, Dotsch, Wigboldus, Langner, and van Knippenberg [43] found that people's mental representations of ethnic outgroup faces are biased to be more criminal and less trustworthy than ethnic ingroup faces and, in related research, participants over-allocated faces appearing criminal-like to an outgroup ethnic category [44]. Such phenomena play out in the political domain as well. In one demonstration, participants rated the representativeness of photographs of biracial political candidates, including Barack Obama, which had been either lightened, darkened, or were un-altered [45]. These researchers found that representativeness ratings tracked with political party affiliation – those who shared the candidate's political party rated the lightened image as most representative, whereas those identifying with an opposing party rated the darkened image as most representative. Importantly, these results remained when controlling for racial attitudes.

In light of such work, it is not altogether surprising that trait ascriptions of faces according to political affiliation are malleable. That is, although targets were seen as more dominant and mature (as well as less likeable and trustworthy) as a function of being categorized as Republicans, the relationship between actual target political affiliation and trait ascriptions was only significant for dominance and maturity, and not likeability or trustworthiness. Further, the relationship between target political categorization and trait ratings was moderated by perceiver ideology. Put more simply, more conservative participants judged faces that appear to be Republicans as likeable and trustworthy, just as previous samples of more liberal participants judged faces that appear to be Democrats as likeable and trustworthy [4].

These results would likely have implications for voting behavior. Olivola et al. [8] found that right-leaning voters favored Republican-looking targets, but that left-leaning voters did not favor Democrat-looking targets. In addition, they found that traits similar to likeability and trustworthiness (i.e., honesty and dependability) did not correlate with the likelihood that a target was categorized as a Republican among a sample of professional politicians. However, they did not report whether this correlation might differ based on perceiver ideology and it is therefore not yet specifically known whether Republican voters are responsive to likeability and trustworthiness in Republican faces versus other dimensions that have been shown to predict voting behavior, such as competence [46]. Perhaps a limitation of our work is that we only used actual target faces with existing political identities. In the future, a more complete investigation into the research question should include the type of data-driven approach described above. That is, using reverse correlation methods, would conservatives and liberals differ on how they visualize Republican and Democrat faces? The current work would support such a prediction. Another limitation is that we were unable to use targets reporting varying levels of conservative and liberal ideology on a continuous scale. Due to the unobtrusive nature in which the targets' photos were acquired, we were only able to gather information regarding political party affiliation. We hope that future research may use non-politician targets with known political ideology as a continuous measure.

It should also be noted that individuals' own political leanings only moderated perceptions of Republican and Democrat faces on certain dimensions. For instance, the correlations between conservatism and perceptions of Republican versus Democrat faces on dominance or maturity were not statistically significant. Rather, Republican faces were rated higher on these traits, unqualified by perceiver ideology. This suggests that there remain some dimensions upon which Republicans and Democrats are widely agreed to differ. It is perhaps sensible that we see moderation by perceiver ideology for traits (i.e., likeability, trustworthiness) that are more clearly positively-valenced whereas less clearly valenced traits (e.g., dominance, facial maturity) may elicit more stable impressions independent of perceiver attitudes. Future work should assess the role of perceiver ideology in assessment of other facial traits, such as competence, attractiveness, and femininity/masculinity. This could help to clarify which types of traits are most malleable to perceiver identities.

The current research advances understanding of the encoding and interpretation of ambiguous social identities. It demonstrates that perceiver identities and motivations are a critical component of social ecological perception [14]. People give off social perceptual affordances that drive perceiver reactions and judgments. Critically, here we see that these affordances are encoded in ways that serve the motivations of the perceiver. Although people can sometimes accurately extract information about others' social identities, such processes may also be systematically biased in self-serving ways.
